# Extensive Neuroparacoccidioidomycosis Lesions with Mild Neurological Manifestations

**DOI:** 10.4269/ajtmh.19-0694

**Published:** 2021-09-27

**Authors:** André Portilho P. O. Freitas, Rodrigo S. Moll Souza, Aloisio Falqueto, Paulo Mendes Peçanha

**Affiliations:** ^1^ School of Medicine, Hospital Universitário Cassiano Antônio Moraes, Universidade Federal do Espírito Santo, Vitória, ES, Brazil;; ^2^ Radiology Department, Hospital Universitário Cassiano Antônio Moraes, Universidade Federal do Espírito Santo, Vitória, ES, Brazil;; ^3^ Infectious Diseases Unit, Hospital Universitário Cassiano Antônio Moraes, Universidade Federal do Espírito Santo, Vitória, ES, Brazil

**Figure 1.**
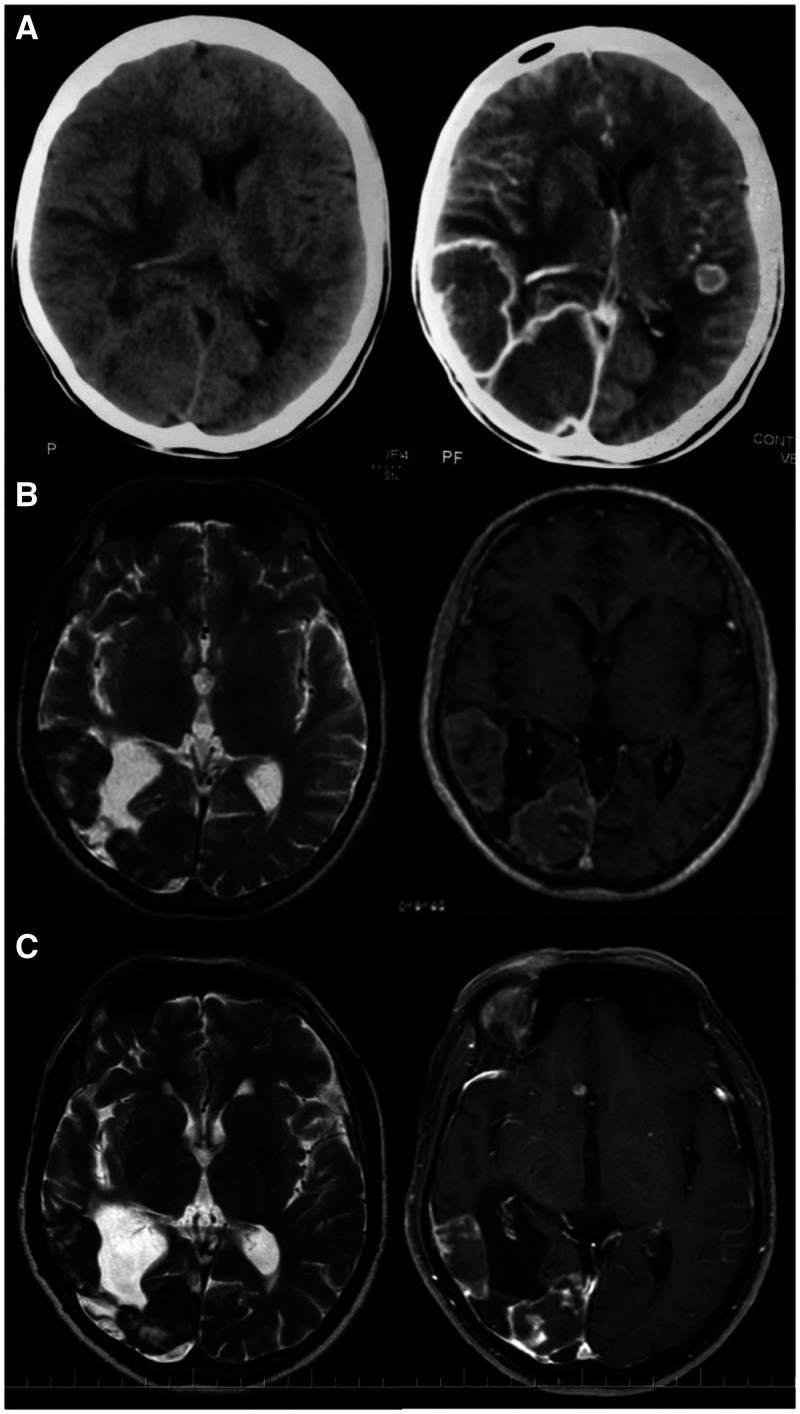
(**A**) Cranial computed tomography showed multiple right tempororoparietal brain lesions. (**B**, **C**) Cranial magnetic resonance images from 2011 and 2016 showed a significant reduction of lesions, but required continuous treatment with cotrimoxazole due to discrete signs of activity.

In November 2009, a 48-year-old male construction worker from a rural area of Espírito Santo state (ES), Brazil, presented with an 8-month history of mild headache, dyspnea, dry cough, fever, weight loss, and left hemifacial paresthesia. Four years earlier, he was diagnosed with chronic pulmonary paracoccidioidomycosis (PCM), confirmed by microbiological analysis and treated with cotrimoxazole 800/160 twice daily for 10 months. He presented with cervical and supraclavicular lymphadenopathy, and there were no pulmonary changes, nor changes on oroscopy or neurological examination. Chest X-ray revealed pulmonary interstitial infiltrates, PCM serology was positive (double immunodifusion title: 1/32), and in bronchoalveolar lavage *Paracoccidioides* sp. was identified, indicating reactivation of PCM. HIV serology and sputum tuberculosis screening were negative. Cranial computed tomography (CT) showed multiple right temporoparietal brain lesions with significant mass effect, displacement of adjacent structures, and contralateral leftward midline shift (Figure 1A). CT of the thorax and abdomen exhibited diffuse parenchymal pulmonary alterations and increased right adrenal gland volume, confirming the diagnosis of chronic multifocal PCM with involvement of the central nervous system (CNS). Treatment was initiated with phenytoin, dexamethasone, and amphotericin B deoxycholate (500 mg) for 10 days, followed by liposomal amphotericin B (2,300 mg) for an additional 10 days. He was discharged in December 2009 using cotrimoxazole and phenytoin. During 6 years of follow-up, the patient showed good clinical evolution. He experienced one episode of generalized tonic-clonic seizures in December 2012, controlled with addition of phenobarbital and valproic acid. Subsequent cranial magnetic resonance images from 2011 and 2016 showed a significant reduction of lesions; however, persistence of discrete signs of activity required continuous treatment with cotrimoxazole (Figure 1B and C).

PCM is the mycosis with the greatest clinical impact in Latin America; 2% of the 10 million infected patients will manifest the disease. The chronic multifocal form occurs primarily among rural workers, with a sex ratio of 15:1 (men:women) and mainly after the fourth decade of life.^1^^,^^2^ Most cases are from Brazil, and the ES state contributes a significant number of these cases. In our retrospective series of 546 cases of PCM, neuroparacoccidioidomycosis occurred in 4.4%.^3^ Other authors have reported incidences ranging from 4% to 27%.^1^^,^^2^^,^^4^ Frequent neurological manifestations include motor signs, intracranial hypertension, seizures, and headache. Multiple granulomatous pseudotumoral masses in the cerebral hemispheres, cerebellum, and brain stem are the most common forms and topographies.^1^^–^^5^ This case highlights the necessity of investigating CNS involvement in patients with multifocal PCM, even with mild neurological symptoms, and also to include Neuroparacoccidiodomycosis as differential diagnosis in tumoral SNC lesions in patients coming from endemic regions.
